# Gooseberry anthocyanins alleviate insulin resistance by regulating ceramide metabolism in high fat diet mice

**DOI:** 10.55730/1300-0152.2668

**Published:** 2023-10-11

**Authors:** Xian TANG, Jun GAO, Jinpeng HUANG, Chenjuan ZHANG, Hongwei LIU, Jie WEI

**Affiliations:** 1 School of Life Science, Liaoning University, Shenyang, Liaoning, 110036, China; 2 Liaoning Academy of Forestry, Shenyang 110032, China

**Keywords:** Gooseberry anthocyanin, insulin resistance, ceramide, high-fat diet mice, molecular docking

## Abstract

**Background/aim:**

Obesity is the fifth largest risk factor of death in the world. The ceramide produced by obesity is closely related to insulin resistance (IR) caused by obesity. At present, the commercially available weight loss products have large side effects and limited therapeutic effects. Therefore, it is particularly important to find effective natural nontoxic products to treat obesity and explore its possible pathways and mechanisms.

**Materials and methods:**

In this paper, a high-fat diet (HFD) mice model was established by intragastric administration of high-fat emulsion to investigate the intervention effect of Gooseberry anthocyanins (GA) on IR in HFD mice. We used molecular docking technology to find the binding sites and binding energy of anthocyanins on CerS6. Real-time PCR was used to detect the effect of GA on the expression of IL-6 and TNF-**α** mRNA in HFD mice. The expression of S1P/Cer signaling pathway in HFD mice with IR was detected by Western Blot.

**Results:**

The results showed that GA could effectively inhibit visceral fat, liver index, the level of TC, TG and the level of LDL-C (p < 0.05), and improved HDL-C, GSH-Px and SOD (p < 0.05). GA decreased the level of insulin sensitivity index from −5.15 to −4.54 and improved insulin sensitivity and IR in HFD mice. The binding energy of anthocyanins on CerS6 was in the range of −8.2 to 5.2 kcal/mol, with low energy parameters and good binding positions. GA could reduce mRNA levels of inflammatory factors IL-6 and TNF-**α** (p < 0.05), inhibit the expression of CerS6, PKC**ζ**, PPAR**γ**, CD36 (p < 0.05), and enhance the expression of SphK2, Akt, p-Akt/Akt, ISR (p < 0.05).

**Conclusion:**

This study investigated the effect and mechanism of GA on reducing ceramide content and reducing IR in mice, and provided an experimental basis for the prevention and treatment of obesity-related diseases.

## 1. Introduction

Obesity is currently a worldwide disease. The inflammatory response, free fatty acids, adipose tissue-specific proteins such as PPAR**γ**, adipocytokines such as ADPN, TNF-**α**, and sphingomyelin substances such as ceramide are closely related to insulin resistance (IR) caused by obesity (Zhou et al., 2009).

**Figure 1:**
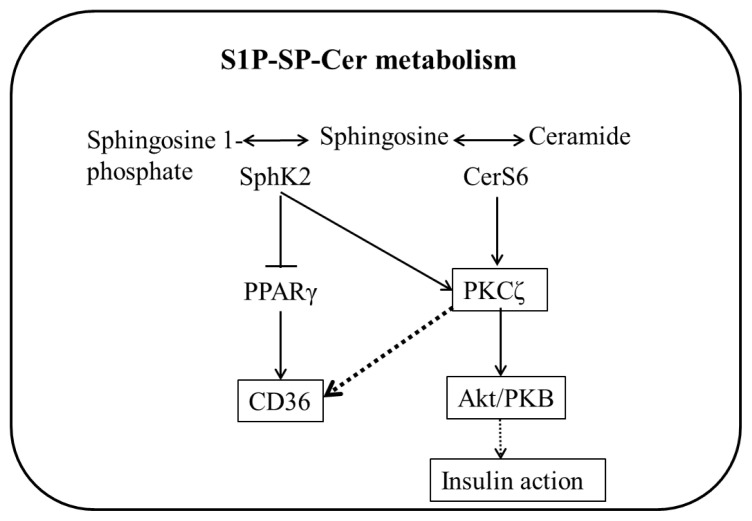
GA restored insulin signaling by suppressing ceramide synthesis: Effect of S1P-SP-Cer metabolism on insulin signal transduction.

IR refers to insulin sensitivity, and the body secretes more insulin to regulate blood glucose at a normal level (Elmadhun et al., 2013). Mainly manifested as obesity; impaired glucose tolerance; serum triglyceride increases and high-density lipoprotein cholesterol decreased; atherosclerosis temperature and hyperuricemia, etc. (Khan and Pessin, 2002). GA on S1P/Cer signaling pathway protein in liver tissue of high-fat diet (HFD) mice S1P-SP-Cer metabolism influences insulin signaling via Akt phosphorylation and CD36 (Figure 1). Studies showed that the prevalence of thyroid dysfunction is quite high in adults who have not yet been diagnosed with diabetes but have IR (KocattFi et al., 2020). Insulin levels increased, and antioxidant capacity weakened, resulting in oxidative stress (Kiraz et al., 2022).

Research showed that ceramide accumulation is linked to obesity, and ceramide was proven to be related to insulin resistance (Chavez and Summers, 2012). Ceramide is the center of sphingomyelin metabolism (Hannun and Obeid, 2002). The accumulation of ceramide in the body will lead to the production of IR (Stratford et al., 2001). Ceramide inhibits insulin signaling, and changes in the ratio of S1P/Cer can lead to apoptosis in myocardial ischemia or ischemia-reperfusion models (Pchejetski et al., 2007). Enzymes that control the synthesis or degradation of sphingolipids in mice have a strong effect on alleviating IR and improving obesity-related lipotoxicity (Holland and Summers, 2008). These studies have shown that drugs that inhibit ceramide synthesis or promote ceramide degradation may be effective targets against IR and related metabolic diseases.

The gooseberry is a Filial generation, the female parent is a European and American hybrid-Oregon, and the male parent is an American species (Ribes Missouriense) (Wei et al., 2018). The fruit is purple-red when fully mature. Its fruit contains rich nutrients, rich amino acids, vitamins, polyphenol and ketones. The main monomer components of GA are: delphinidin 3-O-rutinoside and cyanidin 3-O-rutinoside. Studies showed that anthocyanins have a variety of biological activities, such as antitumoral (Q. Wang et al., 2011), antiviral, antioxidant, antiinflammation, improving metabolic disorders (Wei et al., 2016), inhibit the proliferation of hepatocellular carcinoma cells (Yeh and Yen, 2005), inhibit colon cancer (Wei et al., 2020), age-related neurodegenerative diseases, lowering blood pressure, regulating blood lipids (Kim et al., 2012), improving insulin resistance, effectively delay the aging of mice (Wei et al., 2017) and so on. It has a certain curative effect on cardiovascular disease, diabetes, and other chronic diseases. Gooseberry has good ability for antioxidant, antiinflammation, and scavenging free radicals, preventing osteoporosis, lowering blood pressure, and against radiation and cadmium (J. Gao et al., 2020). Gooseberry showed good antioxidant and antiinflammatory abilities in the previous preexperiment. It is considered that gooseberry has a good development potential and further explores the mechanism of gooseberry in reducing IR in HFD mice. Gooseberry is an excellent variety introduced by Liaoning Province, China. Its yield is very high in the cold weather. The development of gooseberry resources will promote the cultivation of berries and promote the common development of berry agriculture and the food industry.

The purpose of this study is to identify and clarify the mechanism of GA alleviates IR by reducing ceramide in HFD mice. The mice with HFD induced by high-fat emulsion were used as models to study the effect of GA on HFD and its possible mechanism. This provides theoretical support for the treatment of obesity-related diseases in humans and animals.

## 2. Materials and methods

### 2.1. Materials and reagents

Gooseberry fruit provided by Liaoning Academy of Forestry (Shenyang, China). The extraction process of anthocyanin refers to the method used in our laboratory and the anthocyanins were isolated as pure compounds (content > 95%): delphinidin 3-O-rutinoside and cyanidin 3-O-rutinoside. Chloral hydrate was purchased from Beijing Dingguo Biotechnology Co., Ltd. (Beijing, China). Epigallocatechin gallate (EGCG) was purchased from National Pharmaceutical Group Chemical Reagents (Shenyang, China). All other chemicals and solvents used in the experiment were analytical grade.

### 2.2. Animals and treatments

Six-week-old healthy male Kunming (km) mice, weighing 33 **±** 2 g, were provided by Liaoning Changsheng Biotechnology Co., Ltd. (Beijing, China). The animal experiment ethical inspection certificate number was LNU-210316. Fifty male Kunming mice were randomly divided into 5 groups: normal control group (CTL), high fat diet group (HFD), The formula of high-fat emulsion (100 g) is shown in Table 1, high dose GA group (GA 40 mg/kg, HGA), low dose GA group (GA 20 mg/kg, LGA), and positive control group (EGCG 20 mg/kg). GA and EGCG were given to mice by intragastric administration. The entire modeling process lasted 2 months (Li et al., 2020). During the eight-week experiments, the high-fat emulsion was given to mice by the gavage method, and the dose of intragastric administration was 0.2 mL/10 g. The body weight and blood glucose levels of the mice were recorded once a week, and observe their disease status every day. After the dry expectation, the mice fasted overnight, anesthetized by intraperitoneal injection of 0.1 mL of 10% chloral hydrate, and then killed by spinal dislocation. The whole animal experiment process was completed in the Animal Biosafety Laboratory of the College of Life Sciences, Liaoning University.

### 2.3. Molecular dynamic simulations (MD) and docking

The AutoDock vina1.1.2 software package was used for MD simulation. The crystal structure of Human Ceramide synthase 6(CerS6) which was used for this study was downloaded from the Uniprot https://www.uniprot.org/. MGL Tools 1.5.6 software was used for the molecular processing of predocking receptors and ligands. AutoDock vina1.1.2 was used to study the interaction between anthocyanins and CerS6. The three-dimensional structure of anthocyanins was downloaded from Pubchem-Open Chemistry Database (pubchem.ncbi.nlm.nih.gov/substance). AutoDockTools-1.5.6 software was used to prepare CerS6 and anthocyanin molecules before docking. The docking analysis of this research used the Lamarckian genetic algorithm in the AutoDock docking method (Yu et al., 2021).

### 2.4. Serum lipid index

The mice were taken blood from the orbit, and the blood samples were placed in a refrigerator at 4 °C for 0.5 h, frozen and centrifuged, at 3500 rpm/min, centrifuged for 12 min, and the serum was taken. According to the instructions of TC, TG, HDL-C, and LDL-C kits of Beijing Dingguo Biotechnology Co., Ltd. (Beijing, China), the serum lipid indexes of mice were detected, and the absorbance was measured by a microplate reader. The experiment was repeated three times to avoid errors.

### 2.5. Oxidative stress-related enzyme indicators

According to the instructions of SOD and GSH-Px kits (Dingguo Biotechnology Co., Ltd. (Beijing, China), the activities of SOD and GSH-Px in the serum of mice were measured, and the absorbance was measured by microplate reader to reflect the level of oxidative stress. The experiment was repeated three times to avoid errors.

### 2.6. Insulin sensitivity index and resistance index

Fasting insulin (FINS) level and fasting blood glucose concentration (FPG) were measured, cut off food for 8 h ahead of time. Serum insulin levels were measured using the ELISA kit (Dingguo Biotechnology Co., Ltd. (Beijing, China) and blood glucose levels were measured by micro glucometer. The insulin resistance index was expressed by Homeostasis model assessment-Insulin Resistance (HOMA-IR) of the steady-state model. Insulin sensitivity index (ISI) and HOMA-IR were calculated by the following formula (De Luca and Olefsky, 2008).


$$\begin{array}{l} \text{HOMA-IR}=\text{FINS}(\text{mIU}/\text{L})\times \text{FPG}(\text{mmol}/\text{L})/22.5\\ \text{ISI}=\text{ln}[1/\text{FPG}(\text{mmol}/\text{L})\times \text{FINS}(\text{mIU}/\text{L})] \end{array}$$

### 2.7. Histopathological examination of liver tissues

Liver tissues were fixed with 4% formaldehyde solution for 8 h. Tissues were paraffin and sectioned, then stained with hematoxylin and eosin (H&E) (Yang et al., 2020). Tissue sections were observed and photographed under the microscope.

### 2.8. RNA extraction and real-time PCR

Real-time PCR was used to detect the mRNA expression levels of IL-6 and TNF-**α** in liver tissue of mice, referring to KJ Livak‘s method (Livak and Schmittgen, 2001).The primer design and real-time PCR were based on Primer 5.0 software and shown in Table 2. Total RNAs were extracted with Trizol (Tiangen Biotech (Beijing) Co., Ltd.) reagent and purified. Refer to the reaction instructions to synthesize cDNA (Chen et al., 2015). Then PCR amplification was performed.

### 2.9. Western blotting analysis

Western Blot was used to detect the expression of CerS6, p-Akt/Akt, Akt, PKC**ζ**, SphK2, PPAR**γ**, CD36 and ISR in S1P/Cer signaling pathway under IR in HFD mice. The protein samples were separated with 10% SDS-PAGE and transferred to nitrocellulose membrane (Cheng et al., 2015). Tris buffered saline solution containing Tween-20 (Beijing Solarbio Technology Co., Ltd, China) was added to 5% skimmed milk to block the membrane, then added the primary antibody and incubated overnight at 4 **°**C (dilution factor and protein molecular weight was shown in Table 3), and washed the membrane with 1 **×** TBST for three times, 10 min each time. Added the secondary antibody (dilute the secondary antibody with 5% BSA solution) and incubated for 1.5 h at room temperature. Washed the membrane 3 times with 1 **×** TBST, 10 min each time. Expose under the exposure machine, used Image J software to analyze the gray value.

### 2.10. Statistical analysis

Statistical processing results were analyzed with statistical software (GraphPad Prism 8.3.0). The data were analyzed by ANOVO. When p > 0.05, there is no significant difference in the results; when p < 0.05, p < 0.01 or p < 0.001, the result was considered significant.

## 3. Results

### 3.1. Computational analysis of the binding between anthocyanins and CerS6

**Figure 2:**
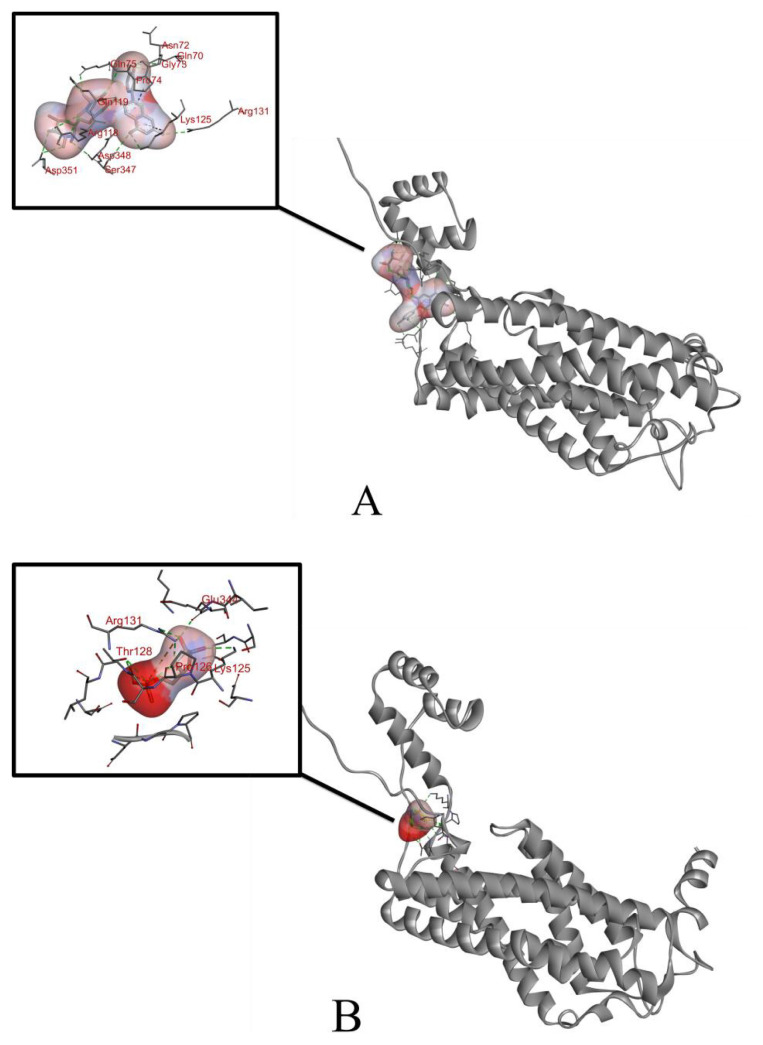
The docking models of two anthocyanins and proteins were analyzed by Autodock software. Displays a panorama of binding modes between cyaniding 3-*O*-rutinoside (A), delphinidin 3-*O*-rutinoside (B), and Cers6. The most conformations of the binding energy are −8.2 and −5.2 kcal/mol.

Docking simulations were used to study two possible anthocyanin binding sites on CerS6. The combination of the model during docking was shown in Figure 2. Anthocyanins may bind to CerS6, and the binding region was shown in Figure 2. From the results of molecular docking analysis, the amino acids composing the binding site of CerS6 and delphinidin 3-O-rutinoside are LYS-125, PRO-126, GLU-344, LYS-125, THR-128. The binding energy of delphinidin 3-O-rutinosid is −5.2 kcal/mol. The binding site of CerS6 and cyaniding3-O-rutinoside are GLN-73, GLN-75, GLN-119, ASN-72, GLY-73, PRO-74, binding energy of cyanidin3-O-rutinoside is −8.2 kcal/mol.

### 3.2. Effects of GA on body weight in HFD mice

**Figure 3:**
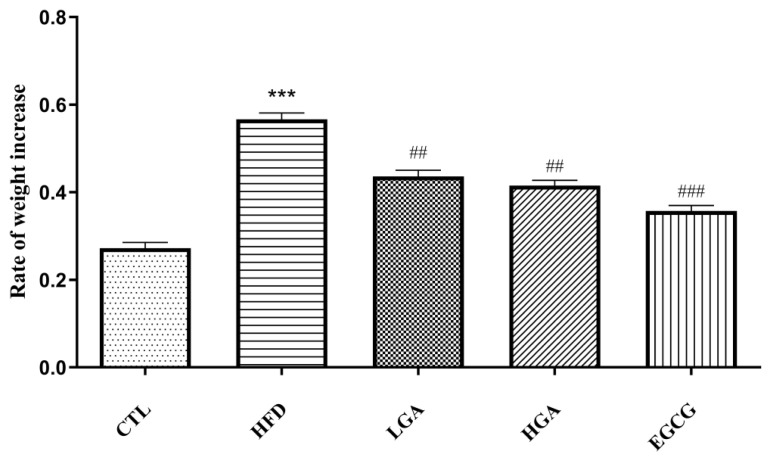
Effect of GA on body weight growth rate of mice. The HFD group compared with the CTL group (***) p< 0.001; Treatment group compared with the HFD group (^##^) p< 0.01, (^###^) p< 0.001.

GA intervention was given to mice at the same time as intragastric administration of high-fat emulsion. The weight changes during the 8-week experimental period are shown in Table 4. From the initial body weight before death, the average body weight of mice in the CTL group was from 31.24 **±** 1.05 g to 44.17 **±** 1.87 g, HFD group from 30.99 **±** 1.14 g to 53.73 **±** 2.24 g. There were significant differences in body weight between the CTL group, HFD group and GA group. It showed that HFD could effectively promote the weight gain of mice, and GA could alleviate this. It could be seen in Figure 3 that the weight growth rate of mice in the HFD group was 1.76 times that of the CTL group, the weight growth rate was significantly increased, the weight growth rate of mice in the LGA and HGA groups was significantly lower than that of in HFD group.

### 3.3. Effects of GA on visceral fat and liver index in HFD mice

**Figure 4:**
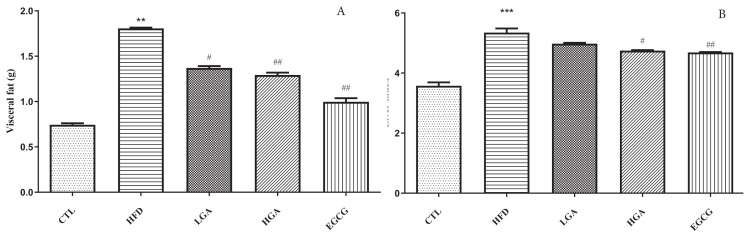
Effect of GA on Visceral fat(A) and Liver index(B) in mice. The HFD group compared with the CTL group(**) p< 0.01,(***) p< 0.001;Treatment group compared with the HFD group(^#^)p < 0.05,(^##^) p< 0.01,(^###^) p< 0.001.

As shown in Figure 4 (A), the visceral fat of the HFD group was 1.81 g, which was significantly higher than that of CTL group (0.74 g). The visceral fat of high and low dose GA group decreased compared with the HFD group, and the visceral fat value of the HGA group decreased to 1.29 g, indicating that high fat emulsion could promote the accumulation of adipose tissue in mice, and GA could slow down the accumulation of adipose tissue in HFD mice.

The liver index of the CTL group was 3.57% as shown in Figure 4(B). The liver index of the HFD group was 5.35%, significantly higher than the CTL group. Compared with the HFD group, the intervention of the EGCG group and HGA group significantly reduced the liver index by 4.75% and 4.69%, and the effect was better than LGA group. It is suggested that high-fat emulsion could increase the liver index of mice, and GA could reduce the liver index of high-fat mice in a dose-dependent manner.

### 3.4. Effect of GA onserum biochemical indexes in HFD mice

**Figure 5:**
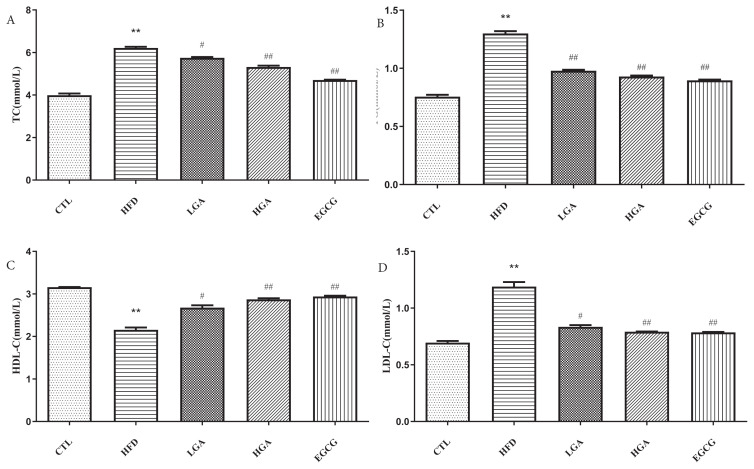
Effect of GA on serum biochemical indexes in mice: TC (A), TG (B), HDL-C (C), LDL-C (D). The HFD group compared with the CTL group(**) p < 0.01;Treatment group compared with the HFD group(^#^)p < 0.05,(^##^) p < 0.01.

As shown in Figure 5, the serum TC and TG levels in the HFD group were higher than those in the CTL group. The expression of TC and TG in the serum of the HGA group and the LGA group were decreased in varying degrees, which indicated that GA could effectively reduce the content of TC and TG in the serum of mice. The level of LDL-C in serum of mice in the HFD group was higher than that in the CTL group, while the level of HDL-C was lower than that in the CTL group. The level of LDL-C in the serum of the HGA and LGA groups showed a decreasing trend compared with that of the HFD group. Compared with the HFD group, the content of HDL-C in the serum of the HGA and LGA groups showed an upward trend. The results showed that GA could reduce the level of LDL-C and increase HDL-C in the serum of HFD mice.

### 3.5. Effects of GA on oxidative stress related enzyme activity in HFD mice

**Figure 6:**
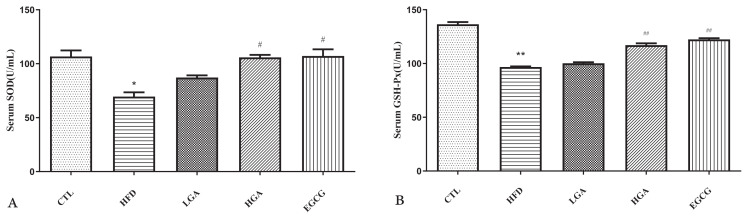
Effects of GA on serum-related enzyme Indexes in mice: SOD activity (A), GSH-Px activity (B) The HFD group compared with the CTL group(*) p < 0.05,(**) p < 0.01; Treatment group compared with the HFD group(^#^) p < 0.05,(^##^) p < 0.01.

As shown in Figure 6, the levels of SOD and GSH-Px are related to oxidative stress. The decrease in SOD and GSH-Px levels indicates decrease in antioxidant capacity, which will lead to an increase in ROS and oxidative stress. The levels of SOD and GSH-Px in the CTL group were 106.69 U/mL and 136.44 U/mL. Oxidative damage occurred in the liver of mice in the HFD group, and the levels of SOD and GSH-Px in the HFD group mice decreased significantly to 69.49 U/mL and 96.74 U/mL. The HGA group returned to 105.86 U/mL and 117.07 U/mL, which were significantly higher than those in the HFD group. The results showed that GA could significantly increase the levels of SOD and GSH-Px in hyperlipidemic mice, and the oxidative stress indexes of the HGA group and EGCG groups were close to those of the CTL group. The results showed that GA could alleviate oxidative stress in mice in a dose-dependent manner.

### 3.6. Effects of GA on insulin sensitivity and insulin resistance index in HFD mice

**Figure 7:**
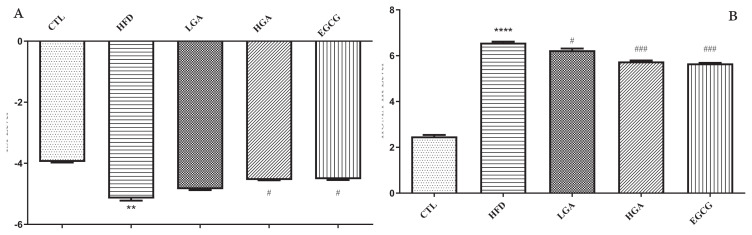
Effect of GA on ISI level (A) and HOMA-IR level (B) staining in mice. The HFD group compared with the CTL group (**) p < 0.01; Treatment group compared with the HFD group (^#^) p < 0.05, (^###^) p < 0.001.

As shown in Figure 7, after 8 weeks of GA intervention, the level of ISI in the HFD group was significantly lower than that in the CTL group. With the increase of GA concentration, the level of ISI in the HGA group increased to-4.54, which increased significantly. It is proved that the intervention of GA could effectively improve the insulin sensitivity of mice. At the same time, the HOMA-IR level of CTL mice was 2.47, and the HOMA-IR level of HFD mice was 6.57, which was significantly higher than that of the CTL group, which proved that HFD mice produced IR successfully. With the increase in GA concentration, the IR index decreased significantly, and the HOMA-IR level of the HGA group decreased to 5.75. The results showed that GA could alleviate IR in mice.

### 3.7. Effect of GA on liver morphology in HFD mice

**Figure 8:**
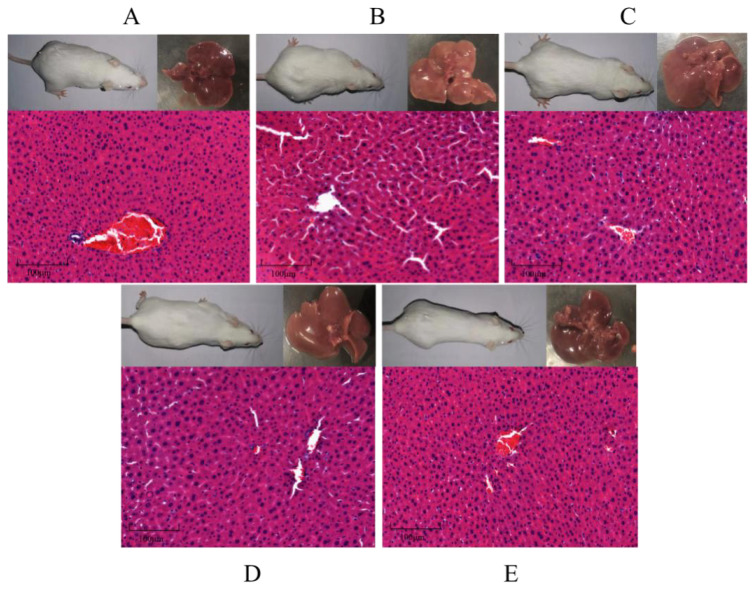
Effects of GA on liver pathology. Histological examination of liver samples from CTL (A), HFD (B), LGA (C), HGA (D), and EGCG (E) group mice. Scale bar: 100 μm.

As shown in Figure 8, the hepatocytes in the CTL group arranged regularly, there were almost no fat vacuoles and steatosis in the visual field, the structure was normal, and there were no obvious pathological changes; in the HFD group, the hepatocytes were accompanied by a certain degree of steatosis, irregular structural arrangement, enlarged hepatocytes, obvious intracellular fat infiltration and fat vacuoles of different sizes, resulting in hepatocyte degeneration. After the intervention of GA, the liver cells of the intervention group were significantly improved compared with the HFD group, and the degree of steatosis of liver cells was alleviated, which was close to the level of the CTL group. The results showed that GA could improve the fat accumulation in the liver of HFD mice.

### 3.8. Effects of GA on the expression of inflammatory cytokines in HFD mice

**Figure 9:**
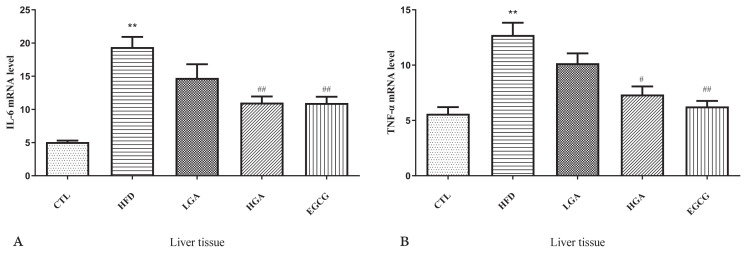
Effects of GA on the expression of inflammatory cytokines in mice. IL-6 mRNA expression in HFD mice(A); TNF-α mRNA expression in HFD mice(B). The HFD group compared with the CTL group (**) p < 0.01; Treatment group compared with the HFD group(^#^)p < 0.05,(^##^) p < 0.01.

It could be seen from Figure 9, the mRNA expression of IL-6 and TNF**α** in liver tissue of HFD group was higher than that of CTL group. Compared with HFD group, the mRNA expression of IL-6 and TNF**α** in EGCG and GA groups showed a downward trend, indicating that GA could effectively down-regulate the mRNA expression of IL-6 and TNF**α** in liver tissue of HFD mice, reduce inflammation in HFD mice.

### 3.9. GA on S1P/Cer signaling pathway protein in liver tissue of HFD mice

**Figure 10:**
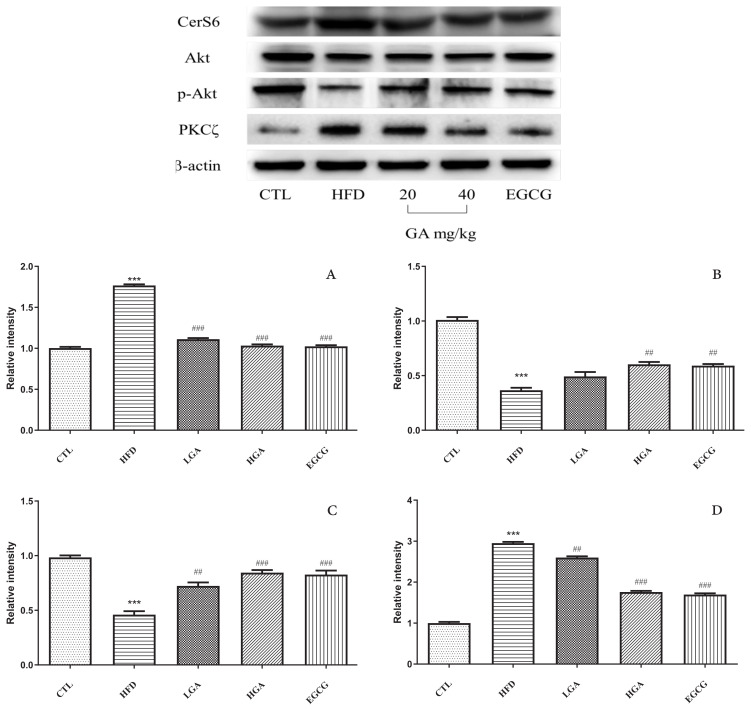
Regulating effect of GA on S1P/Cer signaling pathway in the liver tissue. (A) expression of CerS6 in HFD mice, (B) expression of p-Aktin HFD mice, (C) expression of p-Akt/Aktin HFD mice, (D) expression of PKCζin HFD mice. The HFD group compared with the CTL group(**) p < 0.01, (***) p < 0.001; Treatment group compared with the HFD group(^#^) p < 0.05,(^##^) p < 0.01,(^###^) p< 0.001.

As shown in Figure 10, compared with the CTL group, the expression of CerS6 protein and the downstream protein PKC**ζ** in the liver of the HFD group were significantly increased, and the phosphorylation of the downstream protein Akt and p-Akt/Akt were significantly inhibited. Compared with the HFD group, GA intervention could significantly promote the phosphorylation of Akt and significantly increase the expression of p-Akt/Akt, and significantly inhibit the expression of CerS6 and PKC**ζ** proteins.

**Figure 11:**
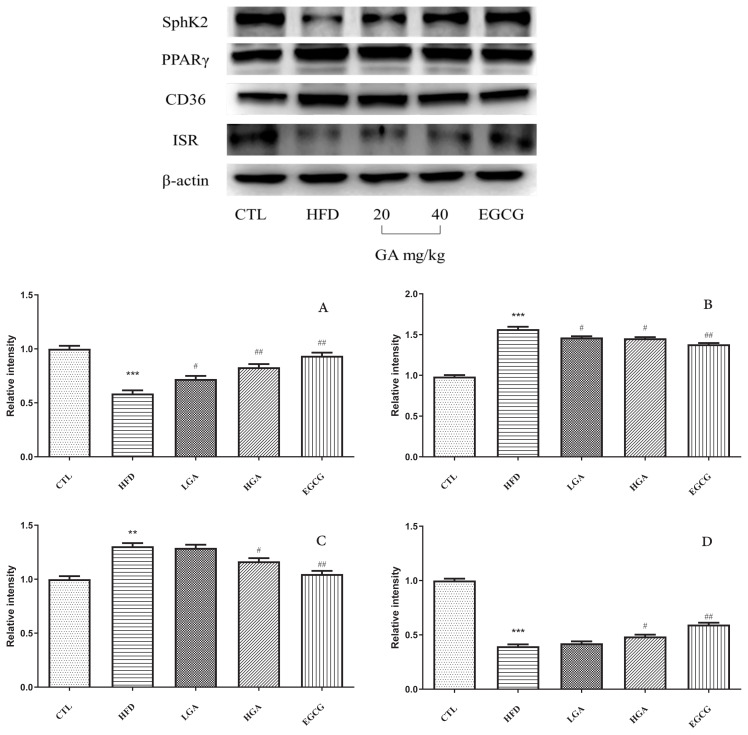
Regulating effect of GA on S1P/Cer signaling pathway in the liver tissue. (A)expression of SphK2 in HFD mice, (B) expression of PPAR**γ** in HFD mice, (C) expression of CD36 in HFD mice, (D) expression of ISR in HFD mice. The HFD group compared with the CTL group(**) p < 0.01, (***) p < 0.001; Treatment group compared with the HFD group(^#^)p < 0.05, (^##^) p < 0.01.

It could be seen from Figure 11, compared with the CTL group, the expression of SphK2 protein in the liver of the HFD group was significantly decreased, and the expression of PPAR**γ** and its downstream proteins CD36 was significantly increased; the expression level of insulin receptor(ISR) was significantly down-regulated. Compared with the HFD group, GA intervention could significantly up-regulate the expression of SphK2 protein and significantly inhibit the expression levels of PPAR**γ** and CD36 proteins, while GA intervention could significantly up-regulate the expression of ISR.

## 4. Discussion

Insulin resistance often occurs in obesity, hypertension, high blood pressure and many cardiovascular diseases (Muniyappa et al., 2007). Anthocyanins could increase insulin sensitivity by increasing the expression of IR, p-IR and p-Akt, then promoting the body’s glucose absorption, thereby achieving the effect of weight loss (Saltiel and Kahn, 2001; Jimenez-Gomez et al., 2014).

Studies have found that the level of ceramide in obese patients is significantly increased, and ceramide plays an important regulatory role in the occurrence of obesity, insulin resistance, atherosclerosis and other diseases (Chaurasia and Summers, 2015). Ceramide blocks the activation of the Akt/PKB pathway in two ways (Chavez et al., 2005). The first is that ceramide mediates the up-regulation of protein phosphatase 2A (PP2A) activity, dephosphorylates Ser473 and Thr308 sites of Akt, leading to the damage of Akt signaling pathway; the second is that atypical PKC**ζ** activated by ceramide reduces the phosphorylation of Akt at Thr34, thereby inhibiting Akt membrane translocation and subsequent activation (Stratford et al., 2001). In our results, GA significantly inhibited the activation of CerS6 in the S1P/Cer signaling pathway, inhibited the expression of ceramide downstream protein PKC**ζ**, and promoted the activation and phosphorylation of Akt. S1P has a positive regulatory effect on cells, while ceramide and sphingosine (SP) have a negative regulatory inhibitory effect on cells. Sphingosine-1-phosphate (S1P) has the exact opposite effect of ceramide and SP, which can be converted into each other before. Sphingosine kinases (SphKs) are the main rate-limiting enzymes that catalyze SP into S1P. The enzyme has two subtypes: SphK1 and SphK2 (Serra and Saba, 2010). SphK2 is the key rate-limiting enzyme that catalyzes the conversion of SP to S1P (Chen et al., 2016). Studies have shown that overexpression of SphK2 can improve liver endoplasmic reticulum stress and reduce liver lipid deposits (Lee et al., 2015). Studies showed a change in the level of intracellular S1P could alter the binding of S1Ps-PPAR**γ** and the transcriptional activity of PPAR**γ**. At the same time, S1P could activate antiapoptotic pathways such as PI3K/Akt through the S1P receptor and sphingosine kinase to improve IR (Riccard and Rivellese, 2004). Through our study, it was found that GA significantly increased the expression level of SphK2 protein in the S1P/Cer signaling pathway, inhibited the expression level of PPAR**γ** and its downstream CD36 protein, and revealed the molecular mechanism of GA in improving insulin resistance in the S1P/Cer signaling pathway.

This study shows the therapeutic role of gooseberry anthocyanins in HFD mice. Compared with medicines, anthocyanins are considered safer and with fewer adverse effects. These results indicate that anthocyanins can improve glucose and lipid metabolism.

## 5. Conclusion

This study showed that GA could improve hepatic steatosis in HFD mice. GA could reduce the level of TG, TC and LDL-C; increased the HDL-C in HFD mice. GA could increase the GSH-Px and SOD activity of liver tissue and alleviate oxidative stress in mice. GA improved IR by inhibiting the S1P/Cer signaling pathway, upregulated the expression ofISR, p-Akt, p-Akt/Akt and SphK2, and reduced the deposition of the expression of CerS6, PPAR**γ**, CD36 and PKC**ζ**. In conclusion, GA could significantly improve insulin resistance in liver tissue. Research showed that GA had protective effects against obesity and IR. GA could inhibit ceramide synthesis in HFD mice and ameliorate IR, which had a certain effect on the prevention and treatment of IR. This study provided a theoretical basis for GA to improve IR in obese mice.

## Figures and Tables

**Table 1:** Experimental mice high-fat emulsion formula.

High-fat emulsion formula	content
Lard	25 g
Tween-80	25 mL
Cholesterol	10 g
Propylthiouracil	1 g
1,2-Propanediol	20 mL
Sodium deoxycholate	2 g
Deionized water	30 mL

**Table 2:** Primer sequences for Ream-Time PCR.

Primer	Upstream primer	Downstream primer
β-actin	5’-AGGCAAACCGTGAAAAGATG-3’	5’-AGGCAAACCGTGAAAAGATG-3’
IL-6	5’TAGTCCTTCCTACCCCAATTTCC-3’	5’TTGGTCCTTAGCCACTCCTTC-3’
TNF-α	5’-CCCTCACACTCAGATCATCTTCT-3’	5’-GCTACGACGTGGGCTACAG-3’

**Table 3:** Dilution factor of protein.

Antibody	Dilution factor	protein molecular weight
Anti-CerS6 antibody	1: 500	44KDa
Anti-PKCζ antibody	1: 500	80KDa
Anti-Akt antibody	1: 1000	60KDa
Anti-p-Akt antibody	1: 1000	60KDa
Anti-SphK2 antibody	1: 300	69KDa
Anti-PPAR**γ** antibody	1: 500	57KDa
Anti-CD36 antibody	1: 500	88KDa
Anti-ISR antibody	1: 500	33KDa
Anti-β-actin antibody	1: 2000	42KDa
HRP GoatAnti-Mouse IgG	1: 5000	-
HRP GoatAnti-Rabbit IgG	1: 5000	-

**Table 4:** Weight changes of mice.

Group		Time week							
	0	1	2	3	4	5	6	7	8
CTL	31.24 ± 1.05	34.93 ± 0.97	36.17± 1.23	37.89± 1.42	40.13± 1.53	41.03± 1.49	42.27± 1.66	43.43± 1.75	44.17± 1.89
HFD	30.99 ± 1.14	34.51±1.02	38.84± 1.81	46.57± 0.97	48.24± 2.05	45.81± 2.06	49.13± 1.59	50.63± 1.85	53.73± 2.24
LGA	30.62 ± 0.87	35.40±1.13	39.03± 1.46	42.73± 1.67	45.56± 1.68	47.51± 2.04	49.20± 1.89	49.97± 2.48	50.57± 2.32
HGA	31.37 ± 0.96	34.79±0.79	37.92± 1.56	42.03± 1.47	44.77± 1.36	46.43± 1.59	47.56± 2.18	48.21± 1.91	49.02± 2.23
EGCG	32.16 ± 0.62	35.41±0.86	38.43± 1.46	41.24± 1.68	44.13± 1.81	45.33± 1.95	46.83± 1.79	47.17± 2.06	47.82± 1.73

* (n = 10, x ± s) The unit is g.

## Data Availability

All data generated or analyzed during this study is included in this published article. Xian Tang, Jun Gao, and Jinpeng Huang-three authors should be regarded as joint first authors.
